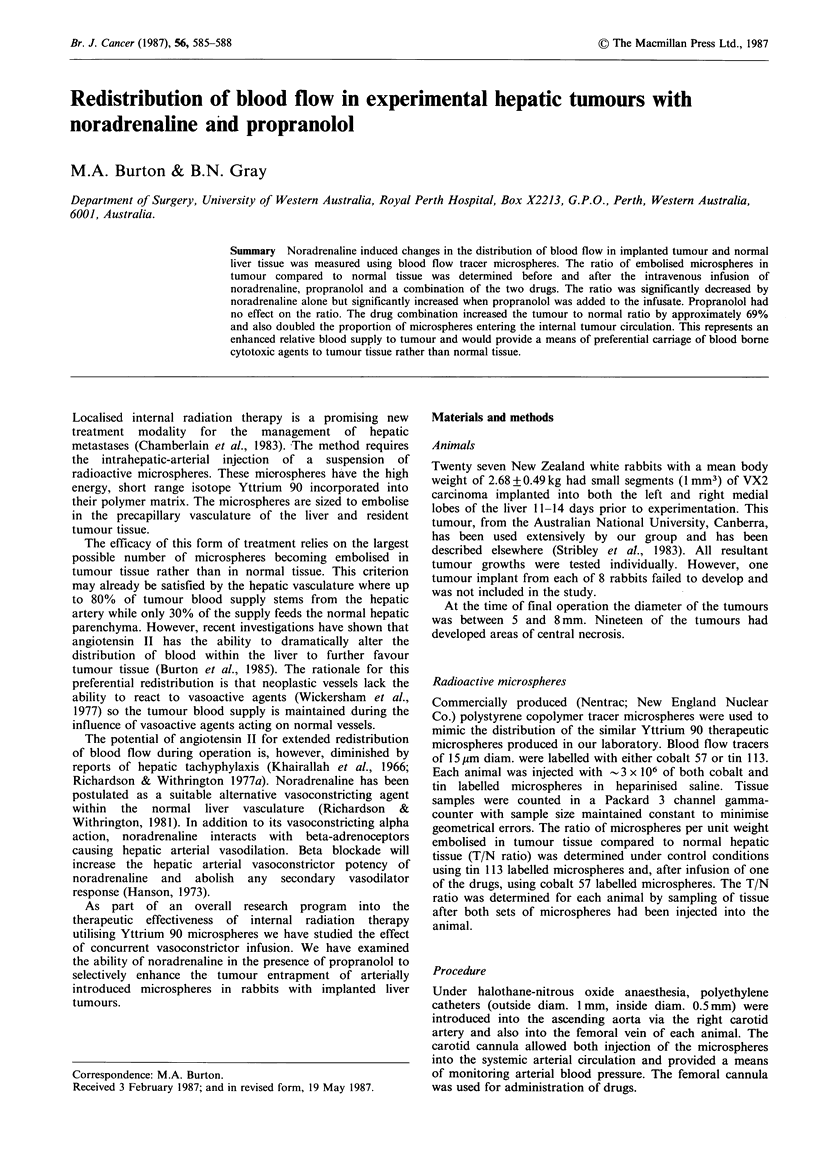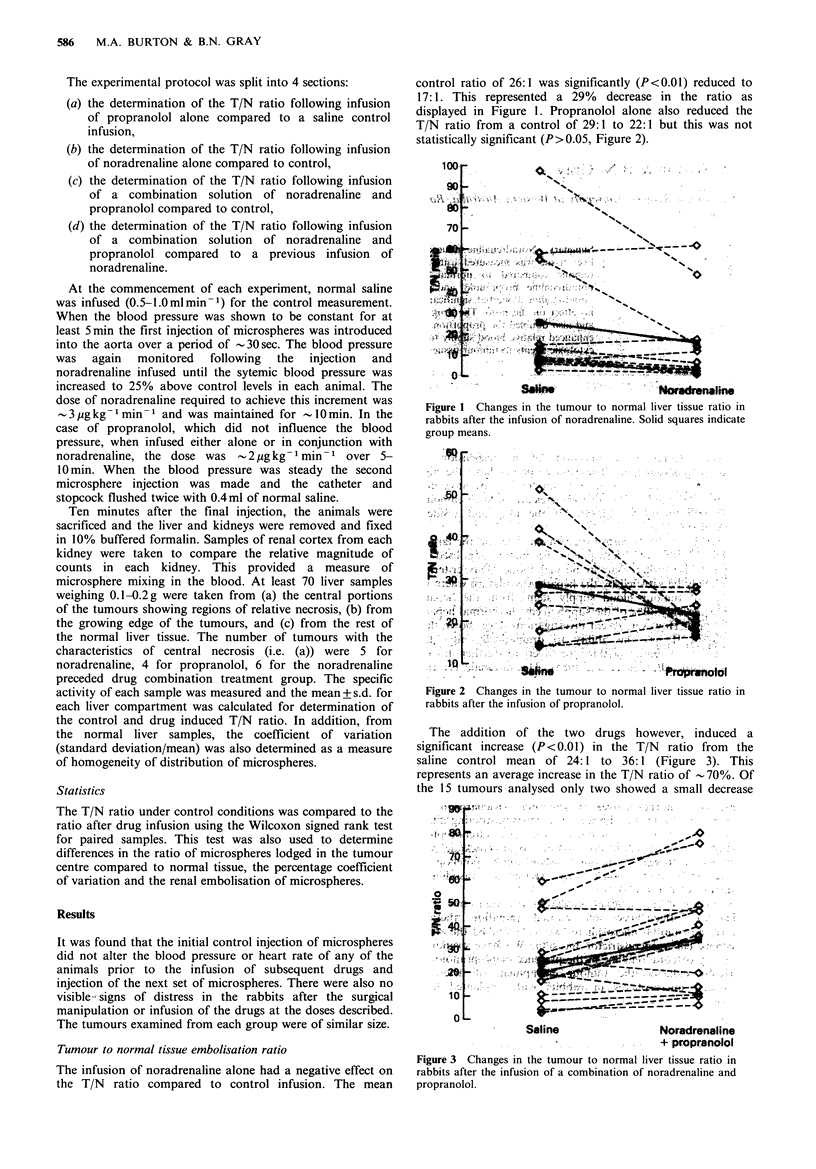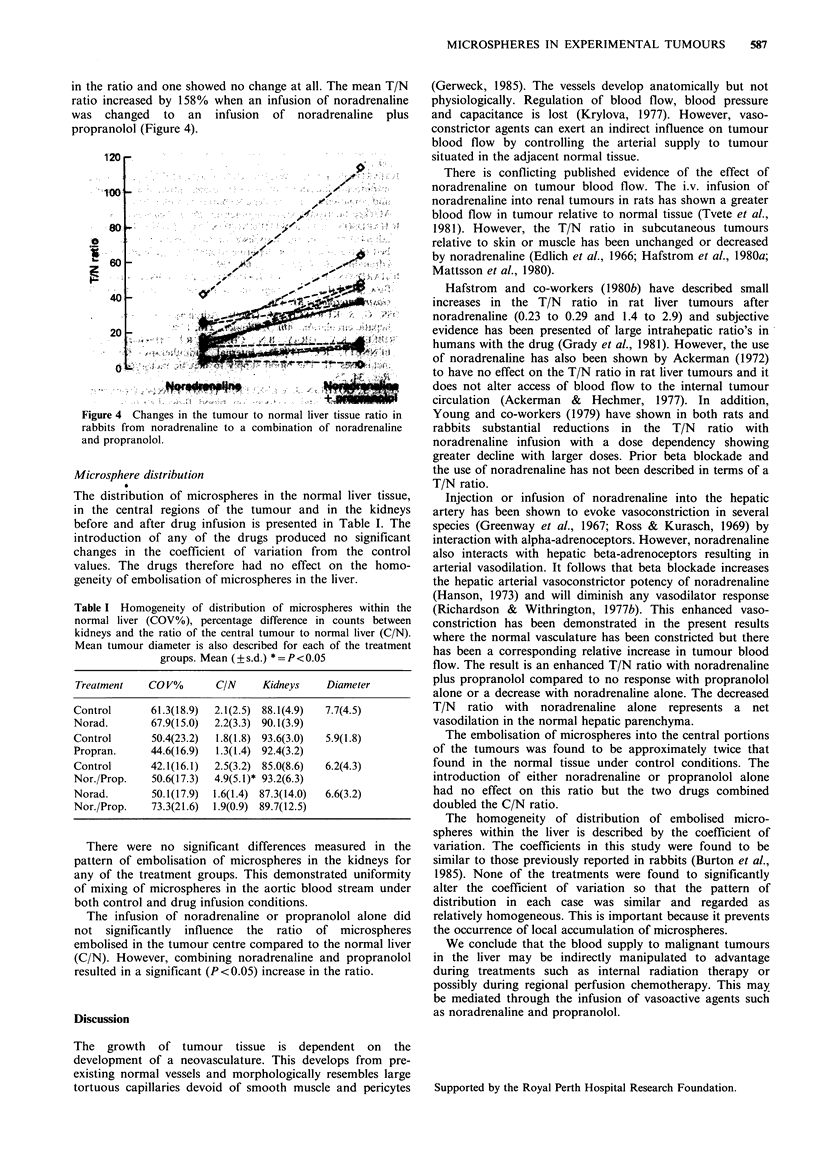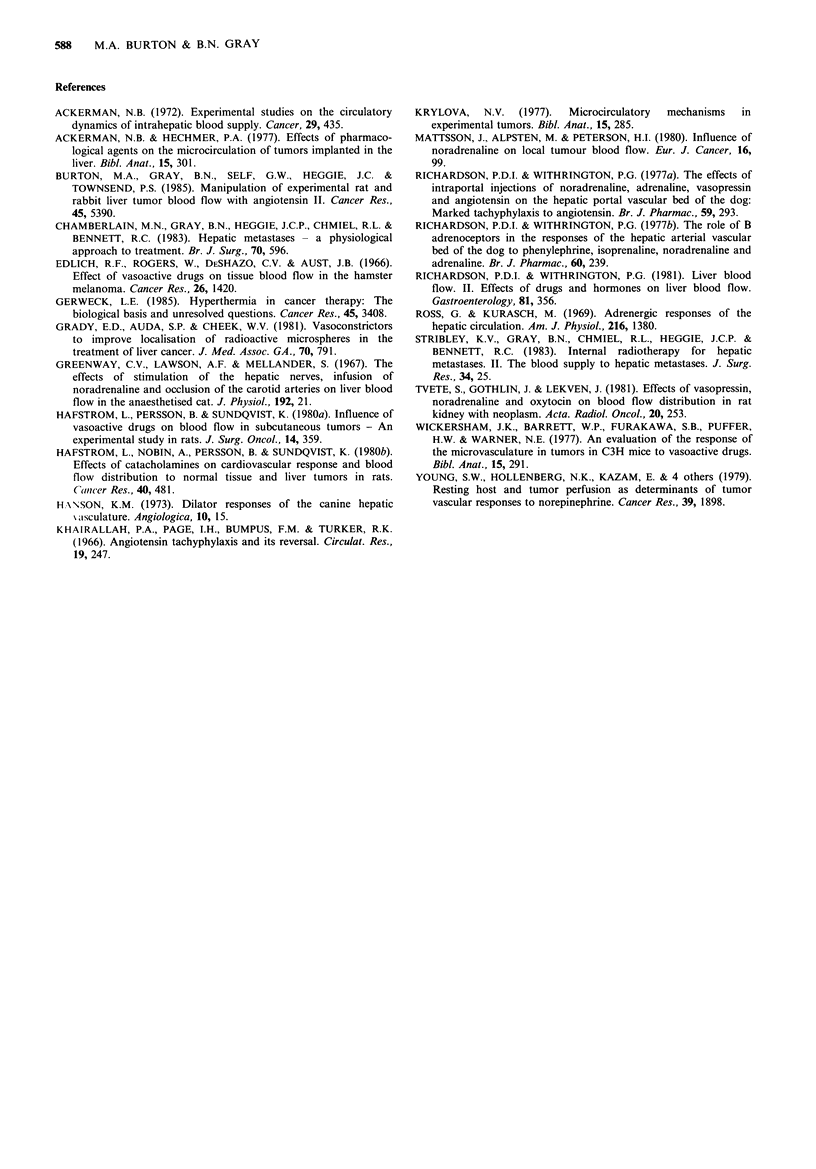# Redistribution of blood flow in experimental hepatic tumours with noradrenaline and propranolol.

**DOI:** 10.1038/bjc.1987.245

**Published:** 1987-11

**Authors:** M. A. Burton, B. N. Gray

**Affiliations:** Department of Surgery, University of Western Australia, Royal Perth Hospital.

## Abstract

Noradrenaline induced changes in the distribution of blood flow in implanted tumour and normal liver tissue was measured using blood flow tracer microspheres. The ratio of embolised microspheres in tumour compared to normal tissue was determined before and after the intravenous infusion of noradrenaline, propranolol and a combination of the two drugs. The ratio was significantly decreased by noradrenaline alone but significantly increased when propranolol was added to the infusate. Propranolol had no effect on the ratio. The drug combination increased the tumour to normal ratio by approximately 69% and also doubled the proportion of microspheres entering the internal tumour circulation. This represents an enhanced relative blood supply to tumour and would provide a means of preferential carriage of blood borne cytotoxic agents to tumour tissue rather than normal tissue.


*Br. J. Cancer* (1987), **56**, 585–588
© The Macmillan Press Ltd., 1987

### Redistribution of blood flow in experimental hepatic tumours with noradrenaline and propranolol

M.A. Burton & B.N. Gray

*Department of Surgery, University of Western Australia, Royal Perth Hospital, Box X2213, G.P.O., Perth, Western Australia, 6001, Australia.*

**Summary** Noradrenaline induced changes in the distribution of blood flow in implanted tumour and normal liver tissue was measured using blood flow tracer microspheres. The ratio of embolised microspheres in tumour compared to normal tissue was determined before and after the intravenous infusion of noradrenaline, propranolol and a combination of the two drugs. The ratio was significantly decreased by noradrenaline alone but significantly increased when propranolol was added to the infusate. Propranolol had no effect on the ratio. The drug combination increased the tumour to normal ratio by approximately 69% and also doubled the proportion of microspheres entering the internal tumour circulation. This represents an enhanced relative blood supply to tumour and would provide a means of preferential carriage of blood borne cytotoxic agents to tumour tissue rather than normal tissue.

Localised internal radiation therapy is a promising new treatment modality for the management of hepatic metastases (Chamberlain *et al.*, 1983). The method requires the intrahepatic-arterial injection of a suspension of radioactive microspheres. These microspheres have the high energy, short range isotope Yttrium 90 incorporated into their polymer matrix. The microspheres are sized to embolise in the precapillary vasculature of the liver and resident tumour tissue.

The efficacy of this form of treatment relies on the largest possible number of microspheres becoming embolised in tumour tissue rather than in normal tissue. This criterion may already be satisfied by the hepatic vasculature where up to 80% of tumour blood supply stems from the hepatic artery while only 30% of the supply feeds the normal hepatic parenchyma. However, recent investigations have shown that angiotensin II has the ability to dramatically alter the distribution of blood within the liver to further favour tumour tissue (Burton *et al.*, 1985). The rationale for this preferential redistribution is that neoplastic vessels lack the ability to react to vasoactive agents (Wickersham *et al.*, 1977) so the tumour blood supply is maintained during the influence of vasoactive agents acting on normal vessels.

The potential of angiotensin II for extended redistribution of blood flow during operation is, however, diminished by reports of hepatic tachyphylaxis (Khairallah *et al.*, 1966; Richardson & Withrington 1977*a*). Noradrenaline has been postulated as a suitable alternative vasoconstricting agent within the normal liver vasculature (Richardson & Withrington, 1981). In addition to its vasoconstricting alpha action, noradrenaline interacts with beta-adrenoceptors causing hepatic arterial vasodilation. Beta blockade will increase the hepatic arterial vasoconstrictor potency of noradrenaline and abolish any secondary vasodilator response (Hanson, 1973).

As part of an overall research program into the therapeutic effectiveness of internal radiation therapy utilising Yttrium 90 microspheres we have studied the effect of concurrent vasoconstrictor infusion. We have examined the ability of noradrenaline in the presence of propranolol to selectively enhance the tumour entrapment of arterially introduced microspheres in rabbits with implanted liver tumours.

Correspondence: M.A. Burton.
Received 3 February 1987; and in revised form, 19 May 1987.

### Materials and methods

### *Animals*

Twenty seven New Zealand white rabbits with a mean body weight of $2.68 \pm 0.49$ kg had small segments (1 mm$^3$) of VX2 carcinoma implanted into both the left and right medial lobes of the liver 11–14 days prior to experimentation. This tumour, from the Australian National University, Canberra, has been used extensively by our group and has been described elsewhere (Stribley *et al.*, 1983). All resultant tumour growths were tested individually. However, one tumour implant from each of 8 rabbits failed to develop and was not included in the study.

At the time of final operation the diameter of the tumours was between 5 and 8 mm. Nineteen of the tumours had developed areas of central necrosis.

### *Radioactive microspheres*

Commercially produced (Nentrac; New England Nuclear Co.) polystyrene copolymer tracer microspheres were used to mimic the distribution of the similar Yttrium 90 therapeutic microspheres produced in our laboratory. Blood flow tracers of 15 $\mu$m diam. were labelled with either cobalt 57 or tin 113. Each animal was injected with $\sim 3 \times 10^6$ of both cobalt and tin labelled microspheres in heparinised saline. Tissue samples were counted in a Packard 3 channel gamma-counter with sample size maintained constant to minimise geometrical errors. The ratio of microspheres per unit weight embolised in tumour tissue compared to normal hepatic tissue (T/N ratio) was determined under control conditions using tin 113 labelled microspheres and, after infusion of one of the drugs, using cobalt 57 labelled microspheres. The T/N ratio was determined for each animal by sampling of tissue after both sets of microspheres had been injected into the animal.

### *Procedure*

Under halothane-nitrous oxide anaesthesia, polyethylene catheters (outside diam. 1 mm, inside diam. 0.5 mm) were introduced into the ascending aorta via the right carotid artery and also into the femoral vein of each animal. The carotid cannula allowed both injection of the microspheres into the systemic arterial circulation and provided a means of monitoring arterial blood pressure. The femoral cannula was used for administration of drugs.

The experimental protocol was split into 4 sections:

(*a*) the determination of the T/N ratio following infusion of propranolol alone compared to a saline control infusion,

(*b*) the determination of the T/N ratio following infusion of noradrenaline alone compared to control,

(*c*) the determination of the T/N ratio following infusion of a combination solution of noradrenaline and propranolol compared to control,

(*d*) the determination of the T/N ratio following infusion of a combination solution of noradrenaline and propranolol compared to a previous infusion of noradrenaline.

At the commencement of each experiment, normal saline was infused (0.5–1.0 ml $min^{-1}$) for the control measurement. When the blood pressure was shown to be constant for at least 5 min the first injection of microspheres was introduced into the aorta over a period of ~30 sec. The blood pressure was again monitored following the injection and noradrenaline infused until the sytemic blood pressure was increased to 25% above control levels in each animal. The dose of noradrenaline required to achieve this increment was ~3 $\mu g\,kg^{-1}\,min^{-1}$ and was maintained for ~10 min. In the case of propranolol, which did not influence the blood pressure, when infused either alone or in conjunction with noradrenaline, the dose was ~2 $\mu g\,kg^{-1}\,min^{-1}$ over 5–10 min. When the blood pressure was steady the second microsphere injection was made and the catheter and stopcock flushed twice with 0.4 ml of normal saline.

Ten minutes after the final injection, the animals were sacrificed and the liver and kidneys were removed and fixed in 10% buffered formalin. Samples of renal cortex from each kidney were taken to compare the relative magnitude of counts in each kidney. This provided a measure of microsphere mixing in the blood. At least 70 liver samples weighing 0.1–0.2 g were taken from (a) the central portions of the tumours showing regions of relative necrosis, (b) from the growing edge of the tumours, and (c) from the rest of the normal liver tissue. The number of tumours with the characteristics of central necrosis (i.e. (a)) were 5 for noradrenaline, 4 for propranolol, 6 for the noradrenaline preceded drug combination treatment group. The specific activity of each sample was measured and the mean ± s.d. for each liver compartment was calculated for determination of the control and drug induced T/N ratio. In addition, from the normal liver samples, the coefficient of variation (standard deviation/mean) was also determined as a measure of homogeneity of distribution of microspheres.

### *Statistics*

The T/N ratio under control conditions was compared to the ratio after drug infusion using the Wilcoxon signed rank test for paired samples. This test was also used to determine differences in the ratio of microspheres lodged in the tumour centre compared to normal tissue, the percentage coefficient of variation and the renal embolisation of microspheres.

### Results

It was found that the initial control injection of microspheres did not alter the blood pressure or heart rate of any of the animals prior to the infusion of subsequent drugs and injection of the next set of microspheres. There were also no visible signs of distress in the rabbits after the surgical manipulation or infusion of the drugs at the doses described. The tumours examined from each group were of similar size.

### *Tumour to normal tissue embolisation ratio*

The infusion of noradrenaline alone had a negative effect on the T/N ratio compared to control infusion. The mean control ratio of 26:1 was significantly ($P<0.01$) reduced to 17:1. This represented a 29% decrease in the ratio as displayed in Figure 1. Propranolol alone also reduced the T/N ratio from a control of 29:1 to 22:1 but this was not statistically significant ($P>0.05$, Figure 2).

**Figure 1** Changes in the tumour to normal liver tissue ratio in rabbits after the infusion of noradrenaline. Solid squares indicate group means.

**Figure 2** Changes in the tumour to normal liver tissue ratio in rabbits after the infusion of propranolol.

The addition of the two drugs however, induced a significant increase ($P<0.01$) in the T/N ratio from the saline control mean of 24:1 to 36:1 (Figure 3). This represents an average increase in the T/N ratio of ~70%. Of the 15 tumours analysed only two showed a small decrease

**Figure 3** Changes in the tumour to normal liver tissue ratio in rabbits after the infusion of a combination of noradrenaline and propranolol.

in the ratio and one showed no change at all. The mean T/N ratio increased by 158% when an infusion of noradrenaline was changed to an infusion of noradrenaline plus propranolol (Figure 4).

**Figure 4** Changes in the tumour to normal liver tissue ratio in rabbits from noradrenaline to a combination of noradrenaline and propranolol.

*Microsphere distribution*

The distribution of microspheres in the normal liver tissue, in the central regions of the tumour and in the kidneys before and after drug infusion is presented in Table I. The introduction of any of the drugs produced no significant changes in the coefficient of variation from the control values. The drugs therefore had no effect on the homogeneity of embolisation of microspheres in the liver.

**Table I** Homogeneity of distribution of microspheres within the normal liver (COV%), percentage difference in counts between kidneys and the ratio of the central tumour to normal liver (C/N). Mean tumour diameter is also described for each of the treatment groups. Mean (±s.d.) * = $P<0.05$

| *Treatment* | *COV%* | *C/N* | *Kidneys* | *Diameter* |
|---|---|---|---|---|
| Control | 61.3(18.9) | 2.1(2.5) | 88.1(4.9) | 7.7(4.5) |
| Norad. | 67.9(15.0) | 2.2(3.3) | 90.1(3.9) | |
| Control | 50.4(23.2) | 1.8(1.8) | 93.6(3.0) | 5.9(1.8) |
| Propran. | 44.6(16.9) | 1.3(1.4) | 92.4(3.2) | |
| Control | 42.1(16.1) | 2.5(3.2) | 85.0(8.6) | 6.2(4.3) |
| Nor./Prop. | 50.6(17.3) | 4.9(5.1)* | 93.2(6.3) | |
| Norad. | 50.1(17.9) | 1.6(1.4) | 87.3(14.0) | 6.6(3.2) |
| Nor./Prop. | 73.3(21.6) | 1.9(0.9) | 89.7(12.5) | |

There were no significant differences measured in the pattern of embolisation of microspheres in the kidneys for any of the treatment groups. This demonstrated uniformity of mixing of microspheres in the aortic blood stream under both control and drug infusion conditions.

The infusion of noradrenaline or propranolol alone did not significantly influence the ratio of microspheres embolised in the tumour centre compared to the normal liver (C/N). However, combining noradrenaline and propranolol resulted in a significant ($P<0.05$) increase in the ratio.

### Discussion

The growth of tumour tissue is dependent on the development of a neovasculature. This develops from pre-existing normal vessels and morphologically resembles large tortuous capillaries devoid of smooth muscle and pericytes (Gerweck, 1985). The vessels develop anatomically but not physiologically. Regulation of blood flow, blood pressure and capacitance is lost (Krylova, 1977). However, vasoconstrictor agents can exert an indirect influence on tumour blood flow by controlling the arterial supply to tumour situated in the adjacent normal tissue.

There is conflicting published evidence of the effect of noradrenaline on tumour blood flow. The i.v. infusion of noradrenaline into renal tumours in rats has shown a greater blood flow in tumour relative to normal tissue (Tvete *et al.*, 1981). However, the T/N ratio in subcutaneous tumours relative to skin or muscle has been unchanged or decreased by noradrenaline (Edlich *et al.*, 1966; Hafstrom *et al.*, 1980*a*; Mattsson *et al.*, 1980).

Hafstrom and co-workers (1980*b*) have described small increases in the T/N ratio in rat liver tumours after noradrenaline (0.23 to 0.29 and 1.4 to 2.9) and subjective evidence has been presented of large intrahepatic ratio's in humans with the drug (Grady *et al.*, 1981). However, the use of noradrenaline has also been shown by Ackerman (1972) to have no effect on the T/N ratio in rat liver tumours and it does not alter access of blood flow to the internal tumour circulation (Ackerman & Hechmer, 1977). In addition, Young and co-workers (1979) have shown in both rats and rabbits substantial reductions in the T/N ratio with noradrenaline infusion with a dose dependency showing greater decline with larger doses. Prior beta blockade and the use of noradrenaline has not been described in terms of a T/N ratio.

Injection or infusion of noradrenaline into the hepatic artery has been shown to evoke vasoconstriction in several species (Greenway *et al.*, 1967; Ross & Kurasch, 1969) by interaction with alpha-adrenoceptors. However, noradrenaline also interacts with hepatic beta-adrenoceptors resulting in arterial vasodilation. It follows that beta blockade increases the hepatic arterial vasoconstrictor potency of noradrenaline (Hanson, 1973) and will diminish any vasodilator response (Richardson & Withrington, 1977*b*). This enhanced vasoconstriction has been demonstrated in the present results where the normal vasculature has been constricted but there has been a corresponding relative increase in tumour blood flow. The result is an enhanced T/N ratio with noradrenaline plus propranolol compared to no response with propranolol alone or a decrease with noradrenaline alone. The decreased T/N ratio with noradrenaline alone represents a net vasodilation in the normal hepatic parenchyma.

The embolisation of microspheres into the central portions of the tumours was found to be approximately twice that found in the normal tissue under control conditions. The introduction of either noradrenaline or propranolol alone had no effect on this ratio but the two drugs combined doubled the C/N ratio.

The homogeneity of distribution of embolised microspheres within the liver is described by the coefficient of variation. The coefficients in this study were found to be similar to those previously reported in rabbits (Burton *et al.*, 1985). None of the treatments were found to significantly alter the coefficient of variation so that the pattern of distribution in each case was similar and regarded as relatively homogeneous. This is important because it prevents the occurrence of local accumulation of microspheres.

We conclude that the blood supply to malignant tumours in the liver may be indirectly manipulated to advantage during treatments such as internal radiation therapy or possibly during regional perfusion chemotherapy. This may be mediated through the infusion of vasoactive agents such as noradrenaline and propranolol.

Supported by the Royal Perth Hospital Research Foundation.

### References

ACKERMAN, N.B. (1972). Experimental studies on the circulatory dynamics of intrahepatic blood supply. *Cancer*, **29**, 435.

ACKERMAN, N.B. & HECHMER, P.A. (1977). Effects of pharmacological agents on the microcirculation of tumors implanted in the liver. *Bibl. Anat.*, **15**, 301.

BURTON, M.A., GRAY, B.N., SELF, G.W., HEGGIE, J.C. & TOWNSEND, P.S. (1985). Manipulation of experimental rat and rabbit liver tumor blood flow with angiotensin II. *Cancer Res.*, **45**, 5390.

CHAMBERLAIN, M.N., GRAY, B.N., HEGGIE, J.C.P., CHMIEL, R.L. & BENNETT, R.C. (1983). Hepatic metastases – a physiological approach to treatment. *Br. J. Surg.*, **70**, 596.

EDLICH, R.F., ROGERS, W., DESHAZO, C.V. & AUST, J.B. (1966). Effect of vasoactive drugs on tissue blood flow in the hamster melanoma. *Cancer Res.*, **26**, 1420.

GERWECK, L.E. (1985). Hyperthermia in cancer therapy: The biological basis and unresolved questions. *Cancer Res.*, **45**, 3408.

GRADY, E.D., AUDA, S.P. & CHEEK, W.V. (1981). Vasoconstrictors to improve localisation of radioactive microspheres in the treatment of liver cancer. *J. Med. Assoc. GA.*, **70**, 791.

GREENWAY, C.V., LAWSON, A.F. & MELLANDER, S. (1967). The effects of stimulation of the hepatic nerves, infusion of noradrenaline and occlusion of the carotid arteries on liver blood flow in the anaesthetised cat. *J. Physiol.*, **192**, 21.

HAFSTROM, L., PERSSON, B. & SUNDQVIST, K. (1980*a*). Influence of vasoactive drugs on blood flow in subcutaneous tumors – An experimental study in rats. *J. Surg. Oncol.*, **14**, 359.

HAFSTROM, L., NOBIN, A., PERSSON, B. & SUNDQVIST, K. (1980*b*). Effects of catacholamines on cardiovascular response and blood flow distribution to normal tissue and liver tumors in rats. *Cancer Res.*, **40**, 481.

HANSON, K.M. (1973). Dilator responses of the canine hepatic vasculature. *Angiologica*, **10**, 15.

KHAIRALLAH, P.A., PAGE, I.H., BUMPUS, F.M. & TURKER, R.K. (1966). Angiotensin tachyphylaxis and its reversal. *Circulat. Res.*, **19**, 247.

KRYLOVA, N.V. (1977). Microcirculatory mechanisms in experimental tumors. *Bibl. Anat.*, **15**, 285.

MATTSSON, J., ALPSTEN, M. & PETERSON, H.I. (1980). Influence of noradrenaline on local tumour blood flow. *Eur. J. Cancer*, **16**, 99.

RICHARDSON, P.D.I. & WITHRINGTON, P.G. (1977*a*). The effects of intraportal injections of noradrenaline, adrenaline, vasopressin and angiotensin on the hepatic portal vascular bed of the dog: Marked tachyphylaxis to angiotensin. *Br. J. Pharmac.*, **59**, 293.

RICHARDSON, P.D.I. & WITHRINGTON, P.G. (1977*b*). The role of B adrenoceptors in the responses of the hepatic arterial vascular bed of the dog to phenylephrine, isoprenaline, noradrenaline and adrenaline. *Br. J. Pharmac.*, **60**, 239.

RICHARDSON, P.D.I. & WITHRINGTON, P.G. (1981). Liver blood flow. II. Effects of drugs and hormones on liver blood flow. *Gastroenterology*, **81**, 356.

ROSS, G. & KURASCH, M. (1969). Adrenergic responses of the hepatic circulation. *Am. J. Physiol.*, **216**, 1380.

STRIBLEY, K.V., GRAY, B.N., CHMIEL, R.L., HEGGIE, J.C.P. & BENNETT, R.C. (1983). Internal radiotherapy for hepatic metastases. II. The blood supply to hepatic metastases. *J. Surg. Res.*, **34**, 25.

TVETE, S., GOTHLIN, J. & LEKVEN, J. (1981). Effects of vasopressin, noradrenaline and oxytocin on blood flow distribution in rat kidney with neoplasm. *Acta. Radiol. Oncol.*, **20**, 253.

WICKERSHAM, J.K., BARRETT, W.P., FURAKAWA, S.B., PUFFER, H.W. & WARNER, N.E. (1977). An evaluation of the response of the microvasculature in tumors in C3H mice to vasoactive drugs. *Bibl. Anat.*, **15**, 291.

YOUNG, S.W., HOLLENBERG, N.K., KAZAM, E. & 4 others (1979). Resting host and tumor perfusion as determinants of tumor vascular responses to norepinephrine. *Cancer Res.*, **39**, 1898.